# Roles for Exosomes in the Pathogenesis, Drug Delivery and Therapy of Psoriasis

**DOI:** 10.3390/pharmaceutics17010051

**Published:** 2025-01-02

**Authors:** Yuchao Chen, Huazhen Liu, Yuming He, Bin Yang, Weihui Lu, Zhenhua Dai

**Affiliations:** 1 Section of Immunology, Guangdong Provincial Academy of Chinese Medical Sciences, 55 Nei Huan Xi Lu, College Town, Guangzhou 510006, China; 2 State Key Laboratory of Dampness Syndrome of Chinese Medicine, the Second Affiliated Hospital of Guangzhou University of Chinese Medicine, Guangzhou 510006, China; 3 Department of Cardiovascular Sciences, College of Life Sciences, University of Leicester, Leicester LE1 9HN, UK

**Keywords:** drug delivery, exosomes, psoriasis, immunoregulation, molecular biology

## Abstract

Psoriasis is a chronic, recurrent and inflammatory skin disease. Although conventional immunosuppressants can ameliorate psoriatic symptoms, it tends to relapse over time. Previous studies have shown that exosomes from both immune and non-immune cells participate in psoriatic immunopathology. The biologically active cargoes in exosomes accelerate psoriasis progression by altering gene profiles and signaling pathways of neighboring cells. On the other hand, exosomes can be utilized as drug delivery platforms for psoriasis treatment. Especially, engineered exosomes may serve as drug delivery systems for effective delivery of proteins, nucleic acids or other drugs due to their low immunogenicity, good stability and ability to fuse with target cells. Therefore, investigation into the mechanisms underlying intercellular communications mediated by exosomes in skin lesions likely helps design drugs for therapy of psoriasis. In this review, we have summarized recent advances in the biogenesis of exosomes and their potential roles in the pathogenesis and treatment of psoriasis and further discussed their challenges and future directions in psoriasis treatment. In particular, this review highlights the immunoregulatory function of exosomes derived from immune or non-immune cells and exosome-based therapeutic applications in psoriasis, including their drug delivery systems. Thus, this review may help accelerate applications of exosomes for drug delivery and treatment of psoriasis.

## 1. Introduction

Psoriasis is a chronic and recurrent inflammatory skin disease, which affects 2–4% of the population worldwide [1]. Based on the clinical manifestations, psoriasis can be classified into four main subtypes, including plaque psoriasis (psoriasis vulgaris), guttate psoriasis, pustular psoriasis and erythrodermic psoriasis. Among them, plaque psoriasis is the most prevalent form, accounting for 80–90% of all cases. Psoriasis is clinically prone to developing erythema, scales and plaque on the scalp, trunk and buttocks [2]. The pathogenesis of psoriasis is a complex process involving interactions between the immune system, environment and genetic predisposition factors; therefore, it still has not been fully elucidated. Currently, the IL-23/IL-17 axis has been considered as the key mechanism underlying the pathogenesis of psoriasis. Activated dendritic cells (DCs) in the dermis can induce the differentiation of Th17 cells through the release of IL-23 and IL-6. Th17 cells, then, induce aberrant proliferation and differentiation of keratinocytes by producing IL-17A/F and IL-22. The abnormal keratinocytes in turn activate DCs by producing proinflammatory cytokines, such as TNF-α, IL-1β, IL-6 and LL-37–self–DNA/RNA complexes [2,3]. Based on the therapeutic targets of the IL-23/IL-17 axis, many biologics have been developed to treat psoriasis. Although these biologics can effectively alleviate psoriasis, skin lesions often recur upon biologics withdrawal [4,5]. Thus, more studies are needed to further understand how immune cells interact with each other or with non-immune cells during the onset and relapse of psoriasis.

Intercellular communication is crucial for the development and maintenance of these diseases. Recently, exosomes have been recognized as important mediators of intercellular communications [6]. Exosomes are small lipid bilayer vesicles secreted by cells, with a diameter range from 30 to 150 nm [7]. They express CD81, CD63 and CD9, and carry many biologically active cargos, such as nucleic acids (DNA, mRNA, miRNA, etc.), specific proteins, lipids and metabolites. These cargos in exosomes are involved in different intercellular communications and regulate cellular biological processes, including metabolism, cell proliferation/differentiation, immune response and signal transduction [8]. Therefore, exosomes, as transport vehicles, not only retain the function of parental cells but also regulate the function of neighboring cells. Studies have shown that exosomes participate in important skin immunopathology, including psoriatic skin inflammation. The exosomes from both immune and non-immune cells in psoriatic lesions play an important role in the pathogenesis of psoriasis [9]. The biologically active cargo in these exosomes can accelerate the development of psoriasis by regulating expression of genes and signaling pathways of neighboring cells [10,11,12]. Hence, further exploration of the mechanisms underlying intercellular communications in psoriatic lesions helps us to understand the pathogenesis of psoriasis. Moreover, exosomes can serve as biomarkers for monitoring the effectiveness of psoriasis treatment because the cargoes or chemical compositions of exosomes change with the progression of psoriasis. More importantly, exosomes can be used as ideal delivery platforms for psoriasis treatment. Previous studies have revealed that exosomes derived from mesenchymal stem cells (MSCs) exert anti-inflammatory effects on psoriasis [13]. Additionally, engineered exosomes can act as delivery systems for efficient delivery of proteins, nucleic acids and/or drugs due to their low immunogenicity, good stability and ability to fuse with target cells.

In this review, we have summarized the biogenesis of exosomes, immunoregulatory function of exosomes derived from immune or non-immune cells and their potential roles in psoriasis as biomarkers, delivery systems or therapeutic agents. We hope this review will help accelerate in-depth studies on their mechanisms of action and applications of exosomes for the treatment of psoriasis.

## 2. The Biogenesis of Exosomes

Exosomes play an important role in intercellular communication under both physiological and pathological conditions. The biogenesis of exosomes is a complex and multistep biological process. Firstly, the plasma membrane is inwards invaginated to produce a primary endosome, which surrounds the biological molecules. Secondly, the multivesicular bodies (MVBs) are generated via inward budding of the endosomal membrane towards the lumen. During this process, proteins and cytosolic components are engulfed and enclosed within the intraluminal vesicles (ILVs). Finally, the MVBs are fused with lysosomes for degradation or the plasma membrane for releasing of ILVs, which are then called “exosomes” [14,15,16,17]. Although the factors determining the fate of MVBs are not fully understood, previous studies have reported that it depends on the amount of cholesterol in MVBs. Cholesterol-rich MVBs tend to secret exosomes, but those with lower cholesterol levels are more prone to lysosomal degradation [18,19].

Previous studies have revealed that the formation of MVBs and sorting of cargoes into exosomes can occur through the endosomal sorting complex required for transport (ESCRT)-dependent and independent pathways [20,21,22]. The ESCRT-dependent pathway is critical for MVB formation, membrane budding and sorting of protein cargoes [17,23]. Four separate protein complexes (ESCRT 0, -I, -II, -III) along with accessory proteins, such as Vps4, VTA1 and ALIX, participate in the ESCRT-dependent pathway [18]. Firstly, ESCRT-0, -I and -II complexes sort ubiquitinated membrane proteins into the invagination in the endosomal delimiting membrane. In addition to recognizing ubiquitinated cargoes, ESCRT-I and ESCRT-II are also involved in membrane germination. Then, the ESCRT-III complex removes the ubiquitin in the shipments and drives vesicle scission, leading to the formation of ILVs. Lastly, Vps4 separates and recycles the ESCRT protein machineries [24,25,26]. Moreover, exosomes can be formed in another way, which is the ESCRT-independent pathway. This pathway involves ceramide- and CD63-dependent processes, heat shock proteins and tetraspanin for sorting cargoes into the exosomes [17,27]. The ceramide can induce membrane budding, resulting in the formation of ILVs [14], while tetraspanin-enriched microdomains are related to the loading of cargo molecules, grouping of particularly targeted receptors, and processing of the essential molecular components into exosomes [28,29].

## 3. The Immunological Function of Exosomes That Are or Are Not Associated with Psoriasis

The pathogenesis of psoriasis is closely related to genetic, environmental and immunological triggers. There are complex interactions between non-immune cells and immune systems in response to triggers [3]. Exosomes can be produced by immune and non-immune cells, such as dendritic cells, macrophages, neutrophils, mast cells, T cells, keratinocytes and adipocytes, which are associated with the pathogenesis of psoriasis. Recent studies have shown that exosomes derived from these cells also can regulate immunity and generate anti-inflammatory responses [30]. Here, we mainly review and discuss the immunological function of exosomes derived from immune or non-immune cells that are or are not associated with psoriasis, as summarized in Table 1.

### 3.1. Immune Cell-Derived Exosomes

#### 3.1.1. Dendritic Cell-Derived Exosomes

Dendritic cells (DCs) are innate immune cells that bridge innate and adaptive immunity by acting as professional APCs and inducing T cell-mediated adaptive immunity. Pathogenic roles for both plasmacytoid DCs (pDCs) and myeloid DCs (mDCs) have been highlighted in psoriasis [2]. In the initial phases of psoriasis development, pDCs are stimulated by self-DNA-LL37 complexes, which are released by keratinocytes (KCs), and then produce IFN-α, which subsequently promotes the maturation and activation of bystander mDCs. Upon activation, mDCs secrete IL-12 and IL-23, which in turn induce naïve T cell differentiation into Th1 and Th17 cells [3].

It has been reported that DC-derived exosomes positively impact the immune system, depending on the maturity of the immune cells [9]. Exosomes released from mature DCs carried MHC-I/II and costimulatory molecules, which were critical for activating T cell-mediated adaptive immunity [31]. Exosomes derived from mature or even immature DCs also promoted the activation of CD4^+^ T cells and the secretion of Th1-cytokines, such as IFN-γ [32]. In addition, DC-derived exosomes could be captured by CD8^+^ DCs with high levels of LFA-1. LFA-1 was a receptor for exosomes to favor antigen transfer between DCs [33]. These findings suggest that exosomes are critical mediators of crosstalk between DCs or DC-Th cells.

#### 3.1.2. Neutrophil-Derived Exosomes

As important innate immune cells, neutrophils can exert antibacterial effects through phagocytosis, degranulation and neutrophil extracellular traps (NETs). An increase in neutrophil migration was found in generalized pustular psoriasis (GPP) [49]. GPP is a type of psoriasis driven by the IL-36 cytokine family [50]. The IL-36-neutrophil loop plays a critical role in the pathogenesis of GPP [2,51]. IL-36 cytokines are primarily expressed by keratinocytes and subsequently broken down by cathepsin, proteinase 3 and elastase in NETs [52]. The biologically active forms of IL-36 can further promote the production of neutrophil chemokines, such as CXCL1, CXCL2 and CXCL8, by keratinocytes, thus attracting more neutrophils to the skin lesions [2,53].

In response to different stimulation, exosomes derived from neutrophils contain different bioactive cargoes, including granule proteins, lipids, microRNA, cytokines and chemokines, which can promote the immune-mediated proinflammatory responses in psoriasis. Exosomes are critical mediators of neutrophil–keratinocyte crosstalk in the pathogenesis of GPP. A previous study found that neutrophils from GPP patients secreted more exosomes than those from control subjects. These exosomes were then rapidly internalized by keratinocytes, which increased the expression of some proinflammatory molecules, such as IL-1β, IL-36G, IL-18, TNF-α and C-X-C motif chemokine ligands (CCLs), in keratinocytes by activating MAPK and NF-κB signaling pathways [12]. Moreover, olfactomedin 4 (OLFM4) in the exosomes released from neutrophils in turn promoted the release of exosomes containing CXCL1, CXCL2, CXCL8 and CCL20 from keratinocytes. These chemokines led to an increase in the migration of neutrophils and other immune cells to the psoriasiform skin lesions [12]. These findings suggest that OLFM4 from neutrophil-derived exosomes is a critical proinflammatory protein. However, it remains unclear how neutrophil-derived exosomes interact with other cells involved in the pathogenesis of psoriasis. Thus, further studies are needed in the near future.

#### 3.1.3. Macrophage-Derived Exosomes

Cutaneous macrophages reside in healthy dermis but may migrate to lymph nodes under the condition of skin inflammation. Macrophages in psoriatic lesions are thought to take part in the development of psoriatic skin inflammation via production of TNF [3,54]. The contents of macrophage-derived exosomes include plasma membrane-associated proteins, cytokines and metabolic enzymes [55]. Yan et al. found that macrophage-derived exosome-shuttled proteinase-activated receptor 3 (Par3) was absorbed by the basal stem cells and enhanced their asymmetric division by activating the Par3/mInsc/LGN signaling pathway, thus leading to psoriatic symptoms in mice [34]. Given that few studies have reported a role for macrophage-derived exosomes in the pathogenesis of psoriasis, more efforts are needed to elucidate their role in the development of psoriasis.

#### 3.1.4. Mast Cell-Derived Exosomes

TNFα is produced primarily by mast cells in psoriatic lesions [56]. Mast cell-derived exosomes contain external antigens and heat shock protein complexes. These exosomes induced immature DCs to upregulate MHC-II, CD80, CD86 and CD40 and to acquire potent capacity of Ag presentation to T cells [35]. Mast cell-derived exosomes also stimulated T cell activation through the OX40L-OX40 pathway, resulting in the induction of Th2 cell differentiation [57]. Furthermore, exosomes released from mast cells reportedly promoted the development of psoriasis. Cheung et al. found that circulating and skin-derived T cells from patients with psoriasis showed an elevated response to phospholipase A2 (PLA2) compared with healthy controls. Exosomes released from IFN-α-induced mast cells could transfer cytoplasmic PLA2 activity to neighboring CD1a-expressing cells, leading to the generation of neolipid antigens. These antigens were subsequently recognized by lipid-specific CD1a-reactive T cells, resulting in the production of IL-17A and IL-22 [36]. These results highlighted a critical role of mast cell-derived exosomes in the pathogenesis of psoriasis, suggesting that inhibition of PLA2 or CD1a may have therapeutic potential for psoriasis.

#### 3.1.5. T Cell-Derived Exosomes

T cells are critical for the pathogenesis of psoriasis [2,56]. Over twenty years ago, psoriasis was originally considered as a Th1-type disease, in which IFN-γ and TNFα were the predominant pathogenic cytokines [58]. But later on with the discovery of the Th17 subset, the IL-23/Th17 axis was thought to be predominant in the pathogenesis of psoriasis [59]. Moreover, impaired function and proliferation of Tregs were found in patients with psoriasis [60,61,62].

It has been shown that T cell-derived exosomes contain TCR, CD3, APO2 ligand, chemokine receptors, CD2, LFA-1 and FasL [63]. T cell-derived exosomes also express CD3, CD2, CD4, CD8, CD11c, CD25, CD69, LFA-1, CXCR4 and GITR [64]. Upon being taken up by different types of cells, T cell-derived exosomes induced a variety of immunoregulatory effects, such as improving APC antigen presentation [65], inhibiting NK cell cytotoxicity [66], regulating DC maturation [67] and enhancing B cell responses and antibody production [68,69]. Moreover, Shefler et al. revealed that T cell-derived exosomes stimulated mast cells to degranulate and release IL-24, which in turn activated keratinocytes in vitro [37]. On the other hand, exosomes released from different T cell subsets may have distinct functions. For example, Treg cell-derived exosomes expressed CD73 that contributed to their suppressive function by converting extracellular adenosine-5-monophosphate to adenosine [38]. Treg-derived exosomes also inhibited proliferation of Th1 cells and their IFN-γ secretion by transferring Let-7d to them [39]. These findings indicate that T cell-derived exosomes have dual immunomodulatory effects, depending on the function of their original cells.

### 3.2. Non-Immune Cell-Derived Exosomes

#### 3.2.1. Keratinocyte-Derived Exosomes

Keratinocytes (KCs) are the main constituents of the epidermis and are critical for the first defense against the invasion of foreign pathogens. However, KCs participate in the development of psoriasis. The terminal differentiation of KCs is incomplete and the proliferation of KC stem cells is dysregulated in psoriasis, resulting in preferential activation and proliferation of the cells. Similar to exosomes derived from other cells, the contents of KC-derived exposomes include chemokines, miRNAs, MHC molecules, etc., which depend on the parent cell status and stimulus [41,70]. KC-derived exosomes are closely related to the development of psoriasis. Treatment of normal KCs with exosomes collected from IL-17-treated KCs upregulated their expression of psoriasis-related genes [40]. Additionally, it was found that exosomes released from environmental pollutant-stimulated keratinocytes induced significant release of proinflammatory cytokines and chemokines in HaCaT cells through the AhR signaling pathway [10].

On the other hand, exosomes derived from KCs also play important roles in cellular crosstalk, thus regulating various cellular functions associated with psoriatic skin inflammation. For example, miRNAs in psoriatic KC-derived exosomes induced T cell polarization and enhanced their differentiation into Th1/Th17 cells, thus promoting psoriasis development [42]. Additionally, KC-derived exosomes altered dendritic cell phenotypes and cytokine production [43]. Jiang et al. also found that KC-derived exosomes activated the NF-κB and p38 MAPK pathways, leading to the release of NETs and production of IL-6, IL-8 and TNF-α by neutrophils. These exosomes further exacerbated psoriatic skin lesions in imiquimod (IMQ)-induced mice [44]. Furthermore, exosomes released from KCs regulated extracellular matrix (ECM) factors in dermal fibroblasts, activated various signaling pathways affecting fibroblast migration, and upregulated the fibroblast gene FGF-2 in a dose-dependent manner [71,72]. In addition, macrophage polarization may be mediated by intercellular communication with keratinocytes. Jiang et al. revealed that leucine-rich α-2-glycoprotein 1 (LRG1)-enriched exosomes isolated from the primary epidermis of psoriatic mice activated macrophages via a TGF-β receptor 1 (TGFβR1)-dependent process, thus accelerating psoriasiform skin lesions [11]. Another study showed that miR-4505 was highly expressed in the skin tissue of patients with psoriasis. Vitamin D receptor-deficient keratinocyte-derived exosomes (Exos-shVDR) containing miR-4505 significantly promoted macrophage proliferation and polarization towards M1 phenotype while inhibiting macrophage apoptosis. These results imply that miR-4505 may be important for the effects of Exos-shVDR on macrophage function to occur [45].

#### 3.2.2. Adipocyte-Derived Exosomes

Metabolic syndrome and obesity are the main complications of psoriasis. Obesity can increase the risk of psoriasis, while many obesity-related proinflammatory factors are involved in the pathogenesis of psoriasis [73]. The adipose tissue was found to regulate immune cell function/metabolism in distant tissues by secreting exosomes containing bioactive molecules [74]. Moreover, adipose tissue from obese mice released more lipid contents via exosomes than that from lean mice [75]. A previous study also revealed that exosomes released by adipose tissue carried adipokines, such as adiponectin, IL-6, monocyte chemoattractant protein-1 and resistin [76]. Many studies have shown that adipose tissue-derived exosomes can enhance inflammatory reactions. For instance, Wei et al. found that adipose tissue-derived exosomes from obese mice induced M1 macrophage polarization through pro-inflammatory miR-155, thus exacerbating intestinal inflammation [46]. Similar studies showed that miR-221-3p-containing exosomes from the adipose tissue of obese mice induced inflammatory responses in perivascular adipose tissue [77]. Additionally, TNF-α-induced adipocyte-derived exosomes increased leukocyte attachment [78]. However, the role of adipose tissue or adipocyte-derived exosomes in the pathogenesis of psoriasis has not been fully elucidated. More studies are needed to clarify their implications in psoriasis.

#### 3.2.3. Fibroblast-Derived Exosomes

Fibroblasts promote wound healing and maintain the integrity of the skin by producing growth factors and extracellular matrix (ECM) components. However, recent studies have revealed that fibroblasts are involved in the pathogenesis of psoriasis. For example, an inflammation-induced subset of papillary fibroblasts promoted aberrant neurite outgrowth and psoriasiform skin inflammation by secreting the ECM protein tenascin-C (TNC) [79]. Additionally, a single cell and spatial sequencing study demonstrated that a subset of SFRP2^+^ fibroblasts in the context of psoriasis contributed to amplification of the immune network through transition to a proinflammatory state. The SFRP2^+^ fibroblasts also expressed cathepsin S, further amplifying inflammatory responses by activating IL-36G in keratinocytes [80]. A recent study found that DC-secreted LGALS9 was received by CD44^+^ dermal fibroblasts, resulting in an increase in ECM expression and basal epidermal cell hyperproliferation in psoriatic skin [81]. Fibroblasts were also involved in the recurrence of psoriasis. Du et al. found that MMP2^hi^ fibroblasts amplified psoriatic inflammation through the CD100-PLXNB2 axis and upregulated CD103 expression in CD8^+^ T cells, thereby enhancing the tissue residency of CD8^+^ T cells [82].

Fibroblast-derived exosomes may also dually regulate keratinocytes. A previous study reported that exosomes derived from senescent fibroblasts accelerated the closure of epidermal keratinocytes and impaired keratinocyte differentiation in vitro [47]. On the other hand, dermal fibroblast-derived exosomes inhibited epidermal hyperplasia and increased the levels of filaggrin and hyaluronic acid synthase 1 (HAS1) by augmenting the expression of peroxisome proliferator-activated receptor α (PPARα) in HaCaT cells [48]. However, it remains unclear how fibroblast-derived exosomes exactly impact psoriasis.

**Figure 1:**
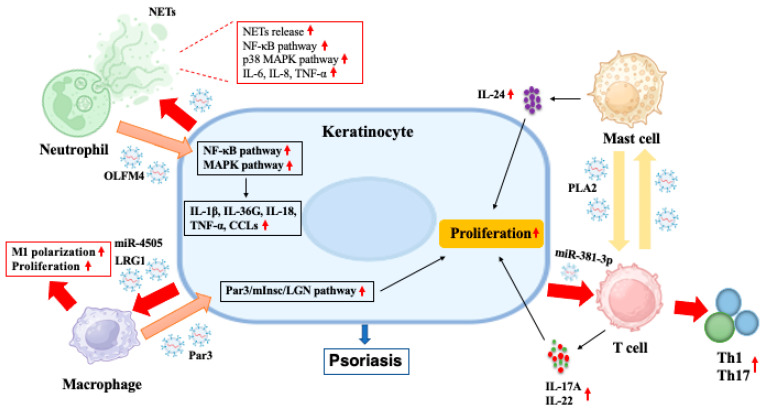
Exosome-mediated communication between immune cells and keratinocytes contributes to the development of psoriasis. Neutrophil-derived exosomes containing olfactomedin 4 (OLFM4) increase the expression of IL-1β, IL-36G, IL-18, TNF-α and CCLs in keratinocytes by activating MAPK and NF-κB pathways. Macrophage-derived exosomes enhance the asymmetric division of basal stem cells by activating the Par3/mInsc/LGN signaling pathway. Mast cell exosomes transfer phospholipase A2 (PLA2) to CD1a-reactive T cells from psoriatic patients, inducing the production of IL-17A and IL-22. T cell-derived exosomes stimulate mast cells to degranulate and release IL-24, which in turn activates keratinocytes. Keratinocyte-derived exosomes containing miR-381-3p promote T cell differentiation into Th1/Th17 cells under psoriatic conditions. These exosomes also increase the release of NETs and production of IL-6, IL-8 and TNF-α in neutrophils by activating the NF-κB and p38 MAPK pathways and promoting the proliferation and M1 polarization of macrophages by delivering miR-4505 and leucine-rich α-2-glycoprotein 1 (LRG1). (Upward, vertical and red arrows denote increasing).

In conclusion, exosomes secreted by either immune or non-immune cells have a variety of immunomodulatory functions, including antigen presentation, cell proliferation and differentiation and pro-inflammatory responses. Among them, exosomes derived from neutrophils, macrophage, mast cells, T cells and keratinocytes are involved in the pathophysiological processes of psoriasis, as shown in Figure 1. In brief, the exosomes from neutrophils or macrophages promoted the secretion of inflammatory cytokines and abnormal proliferation in keratinocytes, respectively. Keratinocyte-derived exosomes, in turn, increased the release of NETs by neutrophils and promoted proliferation and M1 polarization of macrophages. Keratinocyte-derived exosomes also promoted T cell differentiation into Th1/Th17 cells in the context of psoriasis. Additionally, mast cell-derived exosomes activated T cells to produce IL-17A and IL-22, while T cell exosomes in turn stimulated mast cells to release IL-24 in the psoriatic skin lesions.

## 4. Exosome Contents May Serve as Biomarkers for the Pathogenesis and Treatment of Psoriasis

Many biologically active cargoes or contents in exosomes are involved in the pathogenesis and treatment of psoriasis. Therefore, they have been used as a biomarker for predicting and evaluating the effectiveness of psoriasis treatment. Zhang et al. compared the expression of miRNAs in exosomes derived from the plasma of good responders (GRs) and non-responders (NRs) after methotrexate (MTX) therapy. They found that the expressions of miR191-5p and miR-21-5p were positively correlated with the severity of psoriasis. Importantly, they identified four microRNAs, including miR-199a-5p, miR-195-5p, miR196a-5p and miR-1246, which could potentially distinguish between GRs and NRs. These findings indicated that these four miRNAs in exosomes might be good biomarkers to predict the efficacy of MTX [83]. Additionally, Paolino et al. investigated the lipid composition of exosomes derived from the plasma of healthy donors (HD) and psoriatic patients treated with or without ustekinumab, an inhibitor of IL-12/IL23 p40. They found that the phospholipid components of exosomes from the psoriatic patients showed an increase in the levels of phosphatidylcholine (PC), phosphatidylethanolamine (PE), phosphatidylglycerol and lysoPC compared to those from HD. Moreover, therapy with ustekinumab reverted the PE and PC levels in exosomes to those of HD. Thus, the levels of PE and PC in plasma exosomes may be used to assess the response of psoriatic patients to ustekinumab treatment [84].

The biologically active cargoes in exosomes can also be utilized to evaluate the effectiveness of traditional Chinese medicine (TCM) in psoriasis treatment. Lv et al. explored the therapeutic effects of Yangxue Jiedu Soup (YJS) on imiquimod (IMQ)-induced psoriasis-like mice. The results indicated that YJS significantly inhibited Toll-like receptor 4 (TLR4) activation and NF-κB p65 translocation by suppressing the expression of heat shock protein 70 (HSP70) in exosomes isolated from the plasma, thus alleviating the skin lesions and inflammation in the psoriatic mice [85]. Another study reported by Lv et al. confirmed that luteolin, a natural flavonoid ingredient, significantly relieved the lesions and symptoms of psoriasis by reversing the effects of IFN-γ and inhibiting the expression of HSP90α and HSP90β within exosomes [86]. These findings imply that HSP in exosomes mediates the anti-psoriatic effects of TCM and that it may serve as a biomarker for evaluating the therapy of psoriasis. Taken together, the contents of nucleic acids (microRNAs, such as miR-199a-5p and miR-195-5p), lipids, such as PC/PE, and proteins (eg. HSP90α/β) in exosomes derived from the plasma of psoriasis patients or imiquimod-induced psoriatic mice are closely associated with the pathogenesis of psoriasis and their treatment efficacy.

## 5. Exosome-Based Therapeutic Applications in Psoriasis

### 5.1. Exosomes as Therapeutic Agents for Psoriasis Treatment

**Figure 2:**
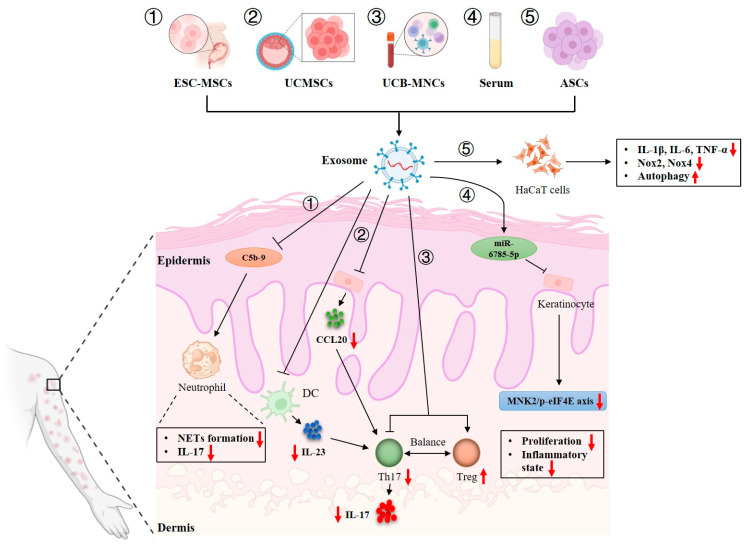
The mechanisms underlying therapeutic effects of exosomes on psoriasis. Embryonic stem cell-derived mesenchymal stem cell (ESC-MSC)-exosomes inhibit C5b-9 complex formation in psoriatic skin, resulting in a reduction in neutrophil extracellular traps (NETs) formation and IL-17 release by neutrophils. Umbilical cord mesenchymal stem cell (UCMSC)-exosomes ameliorate psoriasiform skin inflammation in mice by suppressing the IL-23/IL-17 axis by inhibiting the activation of DCs and CCL20 release from KCs. Umbilical cord blood mononuclear cell (UCB-MNC)-exosomes alleviate acanthosis by restoring the balance of Th17/Treg in IMQ-induced psoriatic mice. Serum-derived exosomes containing miR-6785-5p alleviate psoriasis-like skin lesions by inhibiting the abnormal proliferation and inflammatory state of keratinocytes by interfering with the MNK2/p-eIF4E axis. Adipose tissue-derived stem cell (ASC)-exosomes suppress the production of IL-1β, IL-6 and TNF-α and the expression of Nox2 and Nox4, and they induce autophagy in HaCaT cells. (Upward, vertical and red arrows denote increasing, while downward, vertical and red arrows indicate decreasing).

Recently, exosomes have received wide attention in terms of their potential application in the treatment of autoimmune diseases, including psoriasis [87], due to their immunomodulatory functions and effects (Figure 2).

Previous studies have proven that exosomes from stem cells, such as umbilical cord MSCs (UCMSCs), embryonic stem cell-derived MSCs (ESC-MSCs) and adipose tissue-derived stem cells (ASCs), can alleviate psoriasiform skin inflammation. Zhang and Lai et al. investigated whether ESC-MSC-derived exosomes could alleviate psoriasiform skin inflammation using a mouse model of IMQ-induced psoriasis. They found that ESC-MSC-exosomes were mainly localized in the stratum corneum of the skin because they were prevented from penetrating into the epidermis by the layers of tight junctions found in the stratum granulosum. A topically applied emulsion of ESC-MSC-exosomes significantly inhibited C5b-9 complex formation by CD59, resulting in a reduction in IL-17 released by NETs of neutrophils [13,88]. Additionally, another study examined the therapeutic effects of UCMSC-derived exosomes on psoriasis using a mouse model of IMQ-induced psoriasis. It was found that UCMSC-exosomes expressed CD9, CD63 and CD81. Subcutaneous injection of these UCMSC-exosomes could significantly ameliorate PASI scores and psoriatic symptoms, such as skin thickness, scaling and erythema. Furthermore, UCMSC-exosomes significantly decreased the expression of p-STAT3, IL-17, IL-23 and CCL20 in both psoriatic mice and HaCaT cells. The in vitro experiments also showed that UCMSC-exosomes inhibited IL-23 secretion from DCs. These results suggest that UCMSC-exosomes may ameliorate psoriasiform skin inflammation in mice by suppressing the IL-23/IL-17 axis by inhibiting the maturation and activation of DCs [89]. Additionally, although ASC-derived exosomes were reportedly involved in the pathogenesis of psoriasis, the ASC-exosomes could also decelerate the development of psoriasis. An in vitro study recently found that ASC-exosomes suppressed the production of proinflammatory cytokines (IL-1β, IL-6 and TNF-α) and expression of oxidative stress-related factors (Nox2 and Nox4) and induced autophagy in HaCaT cells. These effects were attributed to the restoration of autophagy of HaCaT cells by ASC-exosomes [90].

In addition to stem cells, exosomes derived from other types of cells have also been reported to exert anti-psoriatic effects. Rodrigues et al. explored the therapeutic potential of umbilical cord blood mononuclear cells (UCB-MNCs)-derived exosomes for psoriasis. They found that UCB-MNC-exosomes could predispose macrophages to become an anti-inflammatory phenotype. Additionally, UCB-MNC-exosomes reduced the proliferation of CD4^+^/CD8^+^ T cells and the release of proinflammatory cytokines by PBMCs. UCB-MNC-exosomes also increased *FOXP3* expression in PBMCs, suggesting that they can restore the immune balance of Th17/Treg. Moreover, UCB-MNC-exosomes significantly reversed acanthosis and decreased the expression of IL-17A and CCL20 in IMQ-induced psoriatic mice. These findings indicate that UCB-MNC-exosomes may be harnessed for the treatment of Th17-driven inflammatory skin diseases [91]. Recently, another study reported that patients’ serum-derived exosomes containing miR-6785-5p could alleviate psoriasis-like skin lesions both in vitro and in vivo. Mechanically, keratinocytes actively took up the serum-exosomes containing miR-6785-5p, which then inhibited the abnormal proliferation and inflammatory state of keratinocytes by interfering with the MNK2/p-eIF4E axis, thus alleviating psoriasis-like inflammatory skin lesions [92].

Finally, drugs or carriers need to penetrate the stratum corneum before exerting anti-psoriasis effects. However, none of above studies explored how exosomes penetrate through the stratum corneum to exert anti-psoriasis effects. According to previous knowledge, there are two main ways for exosomes to penetrate the stratum corneum, which are the intercellular interstitial pathway and cutaneous appendages pathway. Due to the small size (30–150 nm) and the lipid bilayer structure (similar to the structure of cell membranes), exosomes can directly penetrate the intercellular interstitial between keratinocytes and reach the epidermis and dermis. Additionally, exosomes may enter the dermis directly via cutaneous appendages, such as hair follicles, sebaceous glands and sweat glands. However, cutaneous appendages occupy a relatively low proportion of the skin surface area; thus, the numbers of exosomes absorbed through this pathway are limited.

### 5.2. Exosomes as Drug Delivery Systems for Psoriasis Treatment

**Figure 3:**
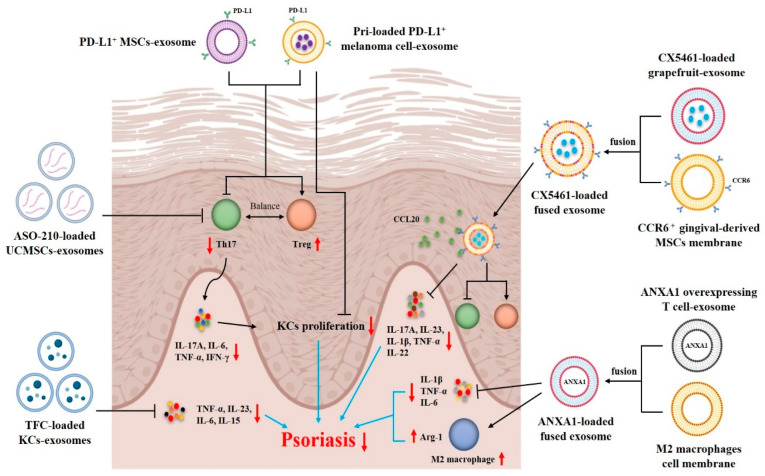
Exosomes serve as drug delivery systems for psoriasis treatment. Umbilical cord mesenchymal stem cell (UCMSC)-derived exosomes improve the delivery efficiency and stability of antisense oligonucleotides of miR-210 (ASO-210). ASO-210-loaded UCMSC-exosomes reduce psoriasis symptoms by suppressing Th17 cells and reducing enrichment of IL-17A, IFN-γ, IL-6 and TNF-α in lesional skin. Compared to free tofacitinib (TFC), TFC-loaded keratinocyte (KCs)-derived exosomes show higher suppressive effects on the expression of TNF-α, IL-23, IL-6 and IL-15 and better therapeutic effects on IMQ-induced psoriasis in mice. Compared to PD-L1^-^ exosomes, PD-L1^+^ MSC-derived exosomes enhance their therapeutic effects on psoriasis by restoring Th17/Treg balance and inhibiting the expression of IL-17A, IL-6 and TNF-α. PD-L1^+^ melanoma cell-derived exosomes can increase pristimerin (Pri) uptake by CD4^+^ T cells, resulting in restoration of the Th17/Treg balance and decrease in abnormal proliferation of KCs. Annexin A1 (ANXA1)-loaded and fused exosomes retain the anti-inflammatory properties of M2 macrophages and ANXA1 protein. They upregulate the expression of M2 macrophage biomarker, arginase-1 (Arg-1), and reduce the levels of IL-1β, IL-6 and TNF-α in lesional skin. Finally, CX5461-loaded and fused exosomes target CCL20-rich inflamed skin through the chemotaxis function of CCR6 expressed on their surface and alleviate IMQ-induced psoriasis by reshaping the Th17/Treg balance and reducing the secretion of IL-17A, IL-23, IL-22, IL-1β and TNF-α. (Upward, vertical and red arrows denote increasing, while downward, vertical and red arrows indicate decreasing).

Currently, exosomes can also be used as delivery systems for psoriasis treatment due to their low immunogenicity, good stability, potent targeting and outstanding ability to fuse with target cells [93,94]. In particular, the engineered exosomes have strong targeting potential and good histocompatibility and thus can deliver proteins, nucleic acids and/or drugs efficiently and accurately. Therefore, exosomes are a promising and novel delivery platform for targeted immunotherapy of psoriasis (Figure 3).

Zhang et al. reported that UCMSC-exosomes could be used to deliver ASO-210 (antisense oligonucleotides of miR-210) for psoriasis treatment. They found that the average size and zeta potential of ASO-210-loaded UCMSC-exosomes were 115 nm and −22.48 mV, respectively, with an average drug loading efficiency of 13.35% and a release efficiency of 94.02% within 48 h. ASO-210-loaded UCMSC-exosomes reduced psoriasis symptoms, exhausted Th17 cells and reduced enrichment of proinflammatory cytokines, such as IL-17A, IFN-γ, IL-6 and TNF-α, in both spleen and skin lesions in vivo. More importantly, UCMSC-exosomes significantly improved the delivery efficiency and stability of ASO-210. Their findings revealed UCMSC-exosomes as promising delivery vehicles of antisense oligonucleotides for psoriasis treatment [95]. Recently, Dehghani et al. chose a probe ultrasound method to load tofacitinib (TFC) into KC-exosomes (TFC-Exo) and further explored the anti-psoriatic effects of TFC-Exo. They demonstrated that TFC-Exo prepared using the probe ultrasound method had the highest drug loading efficiency (30.70%), absolute values of zeta potential (−8.7 mV) and drug release efficiency (67.5%, 24 h). Importantly, compared to free TFC, TFC-Exo exerted higher suppressive effects on the expression of TNF-α, IL-23, IL-6 and IL-15 and better therapeutic effects on IMQ-induced psoriasis in mice. These findings suggest that exosomes can enhance the therapeutic effects of TFC on psoriasis [96].

Engineered exosomes can also be used as delivery systems for psoriasis treatment by increasing their precision targeting. For instance, the PD-1/PD-L1 checkpoint is one of the important pathways for inhibiting immune responses and maintaining immune homeostasis. Binding to PD-1 expressed on the surface of T cells can inhibit their proliferation and production of proinflammatory cytokines [97]. Hence, construction of exosomes with high PD-L1 expression through genetic engineering techniques can enhance their therapeutic effects on psoriasis. Xu et al. constructed PD-L1^+^ MSCs using lentiviral gene transfection technology and collected their exosomes for psoriasis treatment. These exosomes showed a spherical morphology with an average diameter of around 100 nm. In mouse models of IMQ-induced psoriasis, they observed a better therapeutic effect of PD-L1^+^ MSC-exosomes compared to that of PD-L1^-^ MSC-exosomes. PD-L1^+^ MSC-exosomes significantly ameliorated the skin lesions and inflammation by restoring the immune balance of Th17/Treg and inhibiting the expression of proinflammatory cytokines, such as IL-17A, IL-6, IFN-γ, TNF-α and IL-1β. Thus, their findings imply that MSC-derived PD-L1^+^ exosomes can be a potential delivery platform for psoriasis treatment [98]. Recently, Jia et al. chose melanoma cell-derived PD-L1^+^ exosomes for targeted delivery of pristimerin (Pri) to overactive cells in the inflammatory process of psoriasis via PD-1/PD-L1 interaction. They found that the size of Pri-loaded PD-L1^+^ melanoma cell-derived exosomes (Pri@exo) was 89.56 nm, with an ultraviolet absorption peak at 460 nm. Compared to the Pri only group, Pri@exo substantially increased Pri uptake by CD4^+^ T cells and keratinocytes, significantly inhibited the proliferation of Th17 cells and promoted Treg differentiation in a psoriasis-like mouse model. Additionally, Pri@exo also decreased the abnormal proliferation of keratinocytes and production of pro-inflammatory cytokines in psoriatic lesions by alleviating ferroptosis-related changes in the psoriatic skin. These engineered exosomes acted as a “treat-to-target” strategy for psoriasis treatment [99].

The fusion of exosomes with membranes of other cells or nanovesicles is an emerging strategy for psoriasis treatment. The fused vesicles not only maintain the bioactivity of drug-loaded exosomes but also retain the properties of cell membranes or nanovesicles. Wang et al. fused annexin A1 (ANXA1)-overexpressing T cell-derived exosomes with M2 macrophage membranes to obtain ANX1A-loaded fused exosomes. These fused exosomes retained the anti-inflammatory properties of M2 macrophages as well as the macrophage reprogramming potential of ANXA1-overexpressing T cells. They found that the particle size and zeta potential of fused exosomes were 169 nm and –11 mV, respectively. The fused exosomes could be effectively engulfed by macrophages, thus promoting their M2 polarization in vitro. In addition, subcutaneous injection of the fused exosomes significantly upregulated the expression of M2 macrophage biomarker, arginase-1 (Arg-1), and reduced the levels of pro-inflammatory cytokines, including IL-1β, IL-6 and TNF-α in lesional skin, resulting in attenuation of the psoriasis-like skin inflammation [100]. Recently, Huang et al. designed multifunctional fusion nanovesicles that targeted skin lesions for the treatment of autoimmune skin diseases, including psoriasis. Firstly, they loaded CX5461, a small-molecule immunosuppressant with anti-proliferative properties, into grapefruit-derived exosome-like nanovesicles (GEVs) via electroporation. Then, in order to enhance therapeutic efficiency and safety, the exosomes were fused with CCR6^+^ nanovesicles derived from membranes of engineered gingival-derived mesenchymal stem cells (GMSCs) to form FV@CX5461. They found that FV@CX5461 had a spherical-like morphology with the membrane structure, and that the average diameter was about 160 nm, with a zeta potential of around −21 mV. Furthermore, the drug loading efficiency of FV@CX5461was around 18%, while the in vitro drug release rate within 48 h was about 90%. They also confirmed that FV@CX5461 significantly reduced the secretion of proinflammatory factors in HaCaT cells, tempered Th17 cell activation, and induced Treg cell infiltration. Importantly, FV@CX5461 targeted skin lesions and alleviated IMQ-induced psoriasis by reshaping the unbalanced immune microenvironment. They demonstrated that FV@CX5461 not only maintained the bioactivity of GEVs, CX5461 and GMSCs membranes, but also homed to CCL20-rich inflamed skin through the chemotaxis function of CCR6 expressed on the surface [101]. These studies suggest that the fused exosomes may provide potential strategies for the immunotherapy of psoriasis. However, the use of exosomes as a delivery platform for preclinical treatment of psoriasis is still at an early stage, requiring further investigations.

## 6. Clinical Application of Exosomes for Treating Psoriasis

Currently, there are very few clinical studies on exosomes for psoriasis treatment. Most of the studies are still in the preclinical stage. Recently, Mohseni Meybodi et al. have reported the safety and efficacy of MSC-derived exosomes from adipose tissue of healthy donors for treating mild to moderate plaque psoriasis [102]. In this clinical study, twelve patients with plaque psoriasis were divided into three groups and given a single dose of exosomes (50, 100 and 200 μg, respectively). The severity of skin lesions was evaluated, and skin samples were collected pre- and post-treatment for H&E staining and analysis of anti-inflammatory factors. It was found that there were no significant adverse effects seen in patients after the treatment with exosomes. Only two patients experienced minor discomfort. The results showed that the thickness of the lesional skin was significantly decreased in patients receiving 100 and 200 μg doses of exosomes, while erythema and induration were significantly alleviated only in patients receiving a dose of 200 μg. Additionally, the immunohistochemistry results demonstrated that patients receiving 100 and 200 μg of exosomes exhibited a decrease in expression of IL-17 and CD3 and a dramatic increase in Foxp3 in skin lesions. Moreover, the expression of IL-23 and TNF-α in the skin lesions of the patients were significantly decreased, while the expression of IL-10 was increased after treatment with 200 μg of exosomes. These results suggest that 200 μg of exosomes is safe and effective for psoriatic patients in the early phase. These can significantly improve the clinical symptoms and reduce proinflammatory factors in patients with plaque psoriasis. However, the number of patients included in this study is small, and a larger sample size is needed to further verify the effectiveness of MSC-exosomes in psoriasis treatment. In addition, a higher dose or multiple doses may also be considered in future studies.

## 7. Challenges and Future Directions

Despite many benefits of exosome-based therapeutic applications in psoriasis, very few of them are being developed into nanomedicine. Several challenges limit the clinical translation of exosomes in the treatment of psoriasis. Firstly, the existing isolation and separation methods of exosomes exhibit low efficiency, low yields and high costs of scale production, which restricts the large-scale production and application of exosomes. Secondly, the drug loading efficiency of exosomes is less than 20% in most studies of psoriasis treatment. Novel drug loading methods need to be developed for increasing the drug loading efficiency of exosomes to reduce the manufacturing costs. Thirdly, the stability of exosomes in vitro and in vivo needs to be improved. Wang et al. found that the particle size of exosomes significantly increased after storing at −80 °C for 6 months, suggesting the occurrence of fusion between exosomes [100]. Moreover, exosomes may be degraded by enzymes in the inflammatory microenvironment of psoriatic lesions.

The future research directions of exosomes for the treatment of psoriasis should focus on the following aspects. Firstly, further studies using engineered exosomes to target certain subsets of immune cells, especially the IL-23/Th17 axis, as well as their signaling pathways to treat psoriasis, are warranted. Secondly, future studies may focus on exosomes targeting memory T cells to prevent the frequent relapse of psoriasis. Finally, efforts may be made to engineer exosomes that can serve as delivery systems for efficient delivery of specific proteins, nucleic acids and/or small molecular drugs to treat psoriasis.

## 8. Conclusions

In conclusion, our review has provided novel insights into the roles of exosomes in the pathological process, immunoregulation, drug delivery, efficacy monitoring and treatment of psoriasis. Further investigation into the mechanisms underlying intercellular communications mediated by exosomes in psoriatic skin lesions would help us understand the pathogenesis of psoriasis and design drugs for the better treatment of psoriasis. Additionally, engineered exosomes as drug delivery systems may be also promising for the treatment of psoriasis. However, most studies on exosomes for the treatment of psoriasis are still in the animal experimental stage. More studies are needed to optimize the characteristics and functions of exosomes for clinical treatment of psoriasis. More importantly, the source, separation technique and quality control of exosomes should conform to clinical application standards. Overall, exosomes as natural drug carriers combine the advantages of synthetic nanocarriers and cellular communications. Hence, exosomes have great potential and clinical value as drug delivery systems for the treatment of psoriasis. We are optimistic about their future application in the treatment of psoriasis.

## Figures and Tables

**Table 1:** The immunological functions of exosomes derived from immune or non-immune cells.

Source Cells	Contents	Target Cells	Effects	Ref.
Immune cells				
DCs	MHC-II, CD86, ICAM-1	T cells	Stimulating T cells activation	[31]
	MHC-II, CD86, CD9, HLA-DR	CD4^+^ T cells	Activating CD4^+^ T cells and promoting secretion of IFN-γ	[32]
	ICAM-1, MHC-peptide complexes	DCs	Captured by DCs with high levels of LFA-1	[33]
Neutrophils	OLFM4	Keratinocytes	Increasing the expression of IL-1β, IL-36G, IL-18, TNF-α and CCLs in keratinocytes by activating MAPK and NF-κB pathways, which modulate autoinflammation in generalized pustular psoriasis	[12]
Macrophages	Par3	Keratinocytes	Enhancing the asymmetric division of basal stem cells by activating the Par3/mInsc/LGN signaling pathway in psoriatic mice	[34]
Mast cells	HSP60, HSP70	DCs	Inducing immature DCs to upregulate MHC-II, CD80, CD86 and CD40 and to acquire potent Ag-presenting capacity.	[35]
	PLA2	CD1a-expressing cells	Increasing the generation of neolipid antigens in CD1a-expressing cells, which were subsequently recognized by lipid-specific CD1a-reactive T cells from psoriatic patients, thus inducing the production of IL-17A and IL-22	[36]
T cells	Non-specific	Mast cells	Stimulating mast cells to degranulate and release IL-24, which in turn activated keratinocytes	[37]
	CD73	Not specific	Converting extracellular adenosine-5-monophosphate to adenosine, thus promoting interactions with adenosine receptors expressed on target cells	[38]
	Let-7d	Th1 cells	Inhibiting proliferation and IFN-γ secretion of Th1 cells	[39]
Non-immune cells				
Keratinocytes	β-Defensin 2, CCL20, CXCL1, CXCL3, CXCL5, CXCL6	HaCaT cells	Upregulating mRNA expression of endogenous β-Defensin 2 in HaCaT cells	[40]
	CD63, CD9, HSP70, Alix, TSG101		Inducing the release of proinflammatory cytokines (IL-1β, IL-6, IL-8 and TNF-α) and chemokines (CXCL1 and CXCL5) in HaCaT cells through AhR signaling	[10]
	MHC- I, MHC-II	T cells	Enhancing the proliferation of CD4^+^ and CD8^+^ T cells in vitro	[41]
	miR-381-3p	Th1, Th17 cells	promoting T cell differentiation into Th1/Th17 cells under psoriatic conditions	[42]
	Flotillin, ALIX	DCs	Inducing maturation of DCs and their production of IL-6, IL-10 and IL-12	[43]
	CD9, CD63, HSP70	Neutrophils	Increasing the release of NETs and production of IL-6, IL-8 and TNF-α in neutrophils by activating NF-κB and p38 MAPK pathways, thus exacerbating psoriatic skin lesions in mice	[44]
	LRG1	Macrophages	Promoting macrophage polarization through TGFβR1-dependent process, thus accelerating skin lesions in psoriatic mice	[11]
	miR-4505		Enhancing macrophage proliferation and polarization towards M1 phenotype while inhibiting macrophage apoptosis	[45]
Adipocytes	miR-155	Macrophages	Inducing macrophage polarization towards the M1 phenotype	[46]
Fibroblasts	miR-23a-3p	Keratinocytes	Accelerating scratch closure of epidermal keratinocytes and impairing keratinocyte differentiation in vitro	[47]
	Non-specific	HaCaT cells	Inhibiting epidermal hyperplasia and increasing the levels of filaggrin and HAS1 by augmenting the expression of PPARα in HaCaT cells	[48]

## Abbreviations

ANXA1, Annexin A1; Arg-1, Arginase-1; ASO-210, Antisense oligonucleotides of miR-210; ASCs, Adipose tissue-derived stem cells; DCs, Dendritic cells; ECM, Extracellular matrix; ESCRT, Endosomal sorting complex required for transport; ESC-MSCs, Embryonic stem cell-derived mesenchymal stem cells; ILVs, Intraluminal vesicles; IMQ, Imiquimod; KCs, Keratinocytes; MSCs, Mesenchymal stem cells; MVBs, Multivesicular bodies; NETs, Neutrophil extracellular traps; Pri, Pristimerin; TFC, Tofacitinib; UCB-MNCs, Umbilical cord blood mononuclear cells; UCMSCs, Umbilical cord mesenchymal stem cells.
